# Exosomes secreted by human adipose mesenchymal stem cells promote scarless cutaneous repair by regulating extracellular matrix remodelling

**DOI:** 10.1038/s41598-017-12919-x

**Published:** 2017-10-17

**Authors:** Lu Wang, Li Hu, Xin Zhou, Zehuan Xiong, Chenguang Zhang, Hassan M. A. Shehada, Bo Hu, Jinlin Song, Lili Chen

**Affiliations:** 1 Department of Stomatology, Union Hospital, Tongji Medical College, Huazhong University of Science and Technology, Wuhan, 430022 China; 2 College of Stomatology, Chongqing Medical University, Chongqing, 401147 P.R. China

**Keywords:** Skin diseases, Stem-cell research, Engineering

## Abstract

Scar formation is an intractable medical problem that appears after skin wounds have healed. Recent research has shown that exosomes secreted by human adipose mesenchymal stem cells (ASC-Exos) can benefit wound healing. To further explore the therapeutic potential of ASC-Exos, we investigated their effects on mitigating scar formation, and the underlying mechanisms of these effects. We found that intravenous injection of ASC-Exos decreased the size of scars and increased the ratio of collagen III to collagen I in murine incisional wounds. Exosome treatment also prevented the differentiation of fibroblasts into myofibroblasts and increased the ratio of transforming growth factor-β3 (TGF-β3) to TGF-β1 *in vivo*. Additionally, we found that ASC-Exos increased the matrix metalloproteinases-3 (MMP3) expression of skin dermal fibroblasts by activating the ERK/MAPK pathway, leading to a high ratio of MMP3 to tissue inhibitor of matrix metalloproteinases-1 (TIMP1), which is also beneficial for the remodelling of extracellular matrix (ECM). In conclusion, our results demonstrated that ASC-Exos promote ECM reconstruction in cutaneous wound repair by regulating the ratios of collagen type III: type I, TGF-β3:TGF-β1 and MMP3:TIMP1, and by regulating fibroblast differentiation to mitigate scar formation. Therefore, the application of ASC-Exos may be a novel therapeutic approach for scarless wound repair.

## Introduction

Approximately 100 million people annually suffer pain and discomfort caused by scarring^1^. Scarring is associated with depression, social avoidance and disfigurement, and may have devastating consequences for patients, including limitation of movement, poor aesthetic appearance, permanent disability and other effects^2^. Hence, mitigating scar formation is highly desirable for patients. Scar formation is the result of a complex process involving inflammatory, proliferative (the development of granulation tissue) and remodelling phases^3–5^. These three phases are distinct but overlapping. Recent therapeutic approaches include the targeting of inflammatory mediators, epithelial–mesenchymal interactions, gap junctions and connexins, and the transforming growth factor-β (TGF-β) signalling pathway^6^. Therapeutic measures such as surgery, laser therapy and cell therapy have their limitations, which restrain them from being widely introduced into clinical application; no current therapies are optimal and all meet with varying degrees of clinical success^7–9^.

Interestingly, researchers have found that the foetus retains the ability to heal regeneratively, without scars, until late in gestation^10^. Although the molecular mechanisms of foetal wound healing and adult wound healing are incompletely understood, some phenotypic differences have been observed. Scarless wound tissue is characterized by fine reticular collagen, less cross-linking, less inflammation and few myofibroblasts^2^. There are also higher ratios of collagen III to collagen I, TGF-β3 to TGF-β1 and matrix metalloproteinases (MMPs) to tissue inhibitors of MMPs (TIMPs) in scarless wound tissue^11,12^.

To restore the normal architecture of skin and reduce scar formation, investigators have been looking for new ways to mimic foetal wound healing. Recent studies reported that mesenchymal stem cells (MSCs) contributed to wound healing because of their multipotent differentiation ability, high self-renewal capacity, low immunogenicity, and especially their paracrine signalling capacity^13,14^. As a major paracrine factor, exosomes derived from MSCs have attracted the attention of researchers investigating many diseases^15–17^. It has been reported that exosomes might be a novel non-cell therapy and a new mechanism of cell-to-cell communication^18,19^. Previous studies have indicated that exosomes derived from human umbilical cord mesenchymal stem cells increase angiogenesis, suppress myofibroblast differentiation and promote collagen deposition in several wound healing models^20^. Our previous studies have demonstrated that exosomes derived from adipose tissue promote skin wound healing^21^. Accordingly, these data led us to hypothesize that exosomes may be associated with the scarless wound healing.

To evaluate the effects of exosomes on scarless wound healing, we focused on the remodelling of extracellular matrix (ECM), which is one of the most important factors for scar formation^22^. ECM components interact with each other and with the cells that secrete them, regulating cellular metabolism and biological behaviour^23^. Targeting factors involved in ECM production and degradation, such as myofibroblasts, TGF-β, MMPs and TIMPs, may help to improve wound healing and mitigate scar formation^24,25^.

Furthermore, exosomes have been found to active the extracellular regulated protein kinase/mitogen-activated protein kinase (ERK/MAPK) pathway to promote cell proliferation and migration, and enhance angiogenesis^26,27^. MAPKs have been reported to respond to many extracellular signals and to be involved in many cellular behaviours, including wound healing^28,29^. However, whether exosomes modulate the ERK pathway in wound healing and the underlying mechanism of any such effect are still unknown.

In the present study, we hypothesized that exosomes derived from human adipose mesenchymal stem cells (ASC-Exos) could promote ECM remodelling and the scarless healing of cutaneous wounds. To test this hypothesis, we used ASC-Exos to treat mice with dorsal skin incisions *in vivo* or to treat human dermal fibroblasts *in vitro*. We investigated the consequent changes of major factors associated with ECM remodelling. Our results demonstrate that ASC-Exos promote ECM reconstruction by regulating the ratios of collagen type III to type I, TGF-β3 to TGF-β1 and MMP3 to TIMP1, and by regulating fibroblast differentiation to mitigate scar formation during cutaneous wound repair.

## Results

### ASC-Exos mitigated scar formation and increased the ratio of collagen III to collagen I *in vivo*

**Figure 1:**
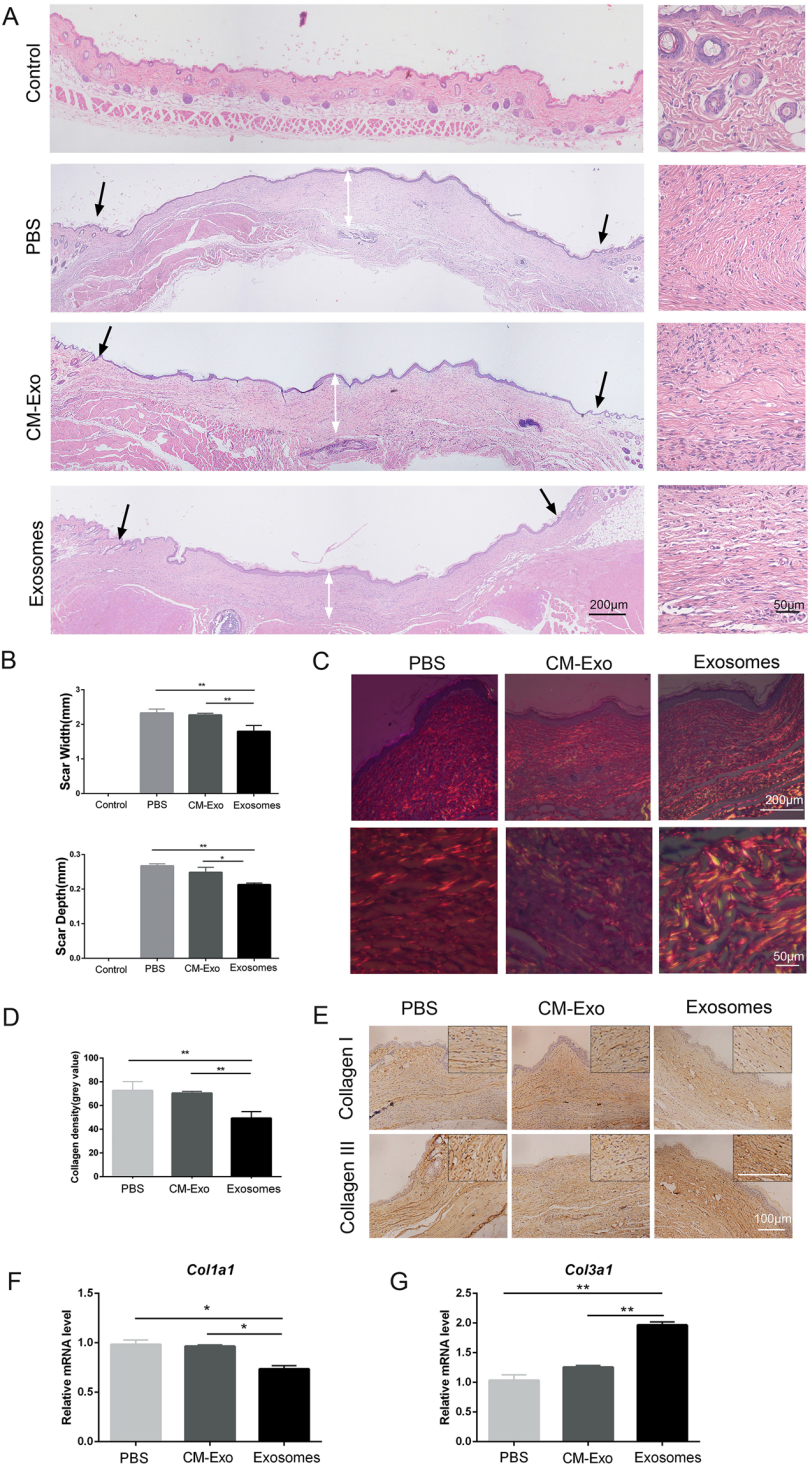
ASC-derived exosomes contributed to reducing scar formation. (**A**) Representative photomicrographs of normal tissue (Control), whole wound sections injected with phosphate-buffered saline (PBS), exosome-free conditioned medium (CM-Exo) or ASC-derived exosomes (Exosomes) at day 21 post-wounding. The black arrows indicate the wound margins, and the white arrows represent the scar depth. Scale bar = 200 µm. (**B**) Quantitative analysis of average scar width and scar depth. (**C**) Picrosirius red staining under polarized light. Scale bar = 200 µm. (**D**) Quantification of collagen density. (**E**) Representative images of immunohistochemical staining of Collagen I and III at day 21 post-wounding. Scale bar = 100 µm. (**F**,**G**) Relative mRNA levels of *Col1a1* and *Col3a1* in wound tissues at day 21 after treatment. Data are shown as mean ± SD; n = 6; *p < 0.05, **p < 0.01, compared with the control groups.

Our previous studies demonstrated that ASC-Exos promoted the healing of cutaneous wounds *in vivo*. In the present study, we investigated the impact of ASC-Exos on scar formation. Histological analysis of haematoxylin and eosin (H&E) -stained full wound sections revealed that exosome treatment attenuated the thickness of the dermal layer and the length of the scar (Fig. 1A,B). In wounds treated with ASC-Exos, the surface of the epidermis was more flattened, and collagen in the dermis was well distributed with less cross-linking. We used picrosirius red staining for further quantitative and qualitative analysis. Under polarized light, bundles of collagen III, which showed greenish/purple-blue birefringence, were apparent in the group treated with ASC-Exos, and the arrangement of collagen was regularly organized in a reticular pattern similar to that of neighbouring unwounded skin. Meanwhile, in the PBS group and the exosome-free conditioned medium (CM-Exo) group, collagen I, which displayed a strong orange-red birefringence, accounted for the majority of dermal collagen (Fig. 1C). In addition, quantification of picrosirius red staining showed a lower collagen density in the exosome-treated group (Fig. 1D). Similarly, the immunohistochemical staining of collagen also showed higher expression of collagen III and modestly lower expression of collagen I in the group treated with ASC-Exos, compared to the PBS group and the CM-Exo group (Fig. 1E). Consistent with the above results, quantitative reverse transcription–polymerase chain reaction (qRT-PCR) assays also suggested that the level of *Col1a1* in skin tissues was lower in response to exosome treatment, while the expression of *Col3a1* increased (Fig. 1F,G). Taken together, these results indicate that the ASC-Exos reduced collagen deposition, mitigated scar formation and increased the ratio of collagen III to collagen I *in vivo*.

### ASC-Exos prevented fibroblasts from differentiating into myofibroblasts, and inhibited granulation tissue formation

**Figure 2:**
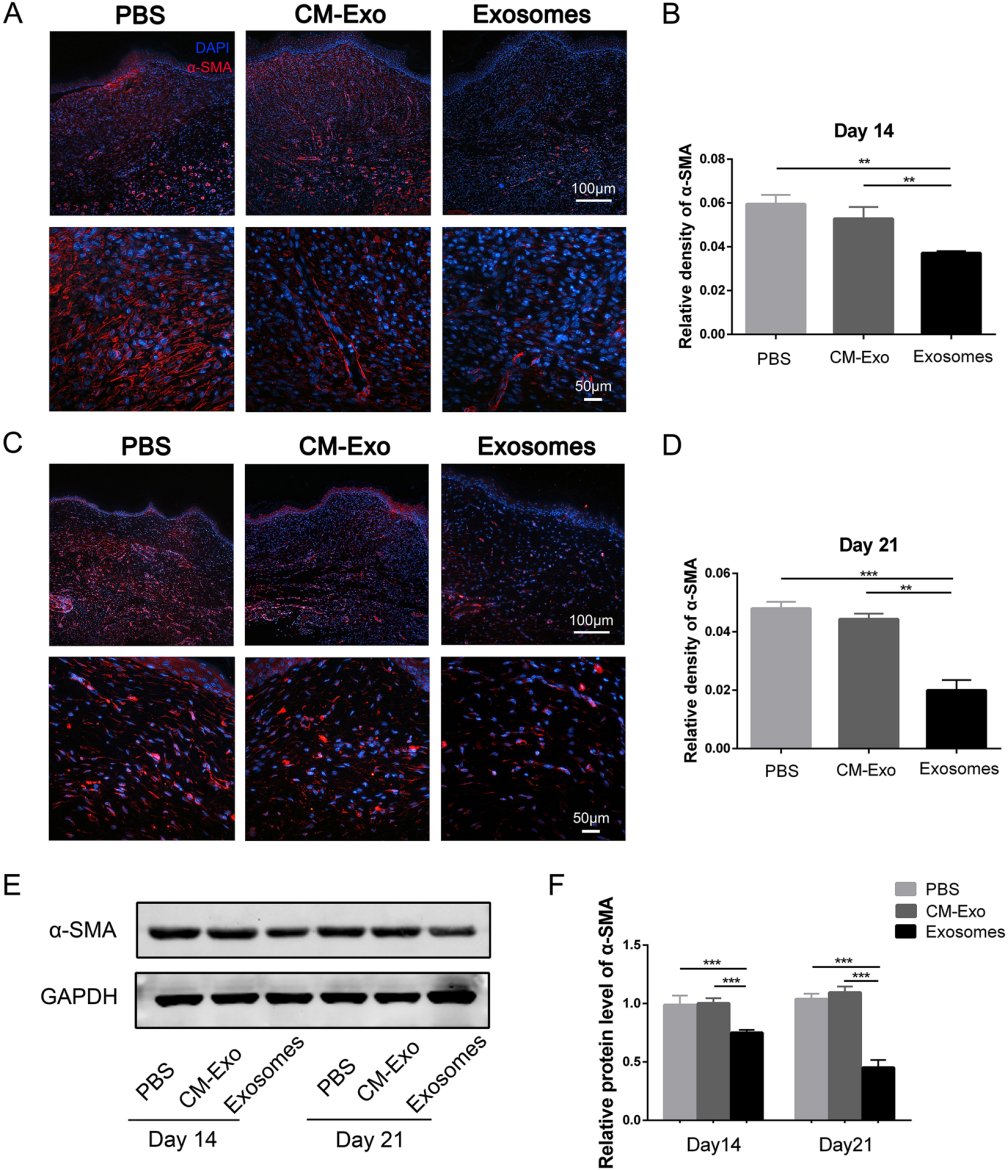
ASC-derived exosomes prevented fibroblasts from differentiating into myofibroblasts, and inhibited granulation tissue formation. (**A**,**B**) Representative immunofluorescence images and analysis of the relative density of α-SMA in wound sections at 14 days following exosome treatment. DAPI, 4,6-diamidino-2-phenylindole. Scale bar = 100 µm. (**C**,**D**) Representative immunofluorescence images and analysis of the relative density of α-SMA in wound sections at 21 days following exosome treatment. Scale bar = 100 µm. (**E**) Western blot analysis of the expression of α-SMAinwound sections at 14 days and 21 days following exosometreatment. (**F**) Densitometric analysis of the western blot bands. Results are presented as mean ± SD; n = 3; **p < 0.01, ***p < 0.001, compared with the control groups.

In the proliferative phases of wound healing, fibroblasts are activated and differentiate into myofibroblasts, which are observed in the granulation tissue of healing wounds^30,31^. Myofibroblasts synthesize ECM components and promote wound contraction. To further evaluate the differentiation of fibroblasts, we investigated the expression of alpha-smooth muscle actin (α-SMA) in wound tissue. α-SMA is a known marker of myofibroblasts and confers them with high contractility, which contributes to scar formation. Immunofluorescent staining revealed that the expression of α-SMA was significantly decreased at day 14 and 21 after exosome treatment, indicating that exosome treatment suppressed the differentiation of fibroblasts and reduced the number of myofibroblasts residing in the wound bed (Fig. 2A–D). In addition, the decreased level of α-SMA in the exosome-treatedgroup was further confirmed by western blot analysis (Fig. 2E,F). Overall, these results indicate that the ASC-Exos prevented fibroblasts from differentiating into myofibroblasts and inhibited granulation tissue formation.

### ASC-Exos increased the ratio of TGF-β3 to TGF-β1 ***in vivo*** and accumulatedin the vicinity of the wound

**Figure 3:**
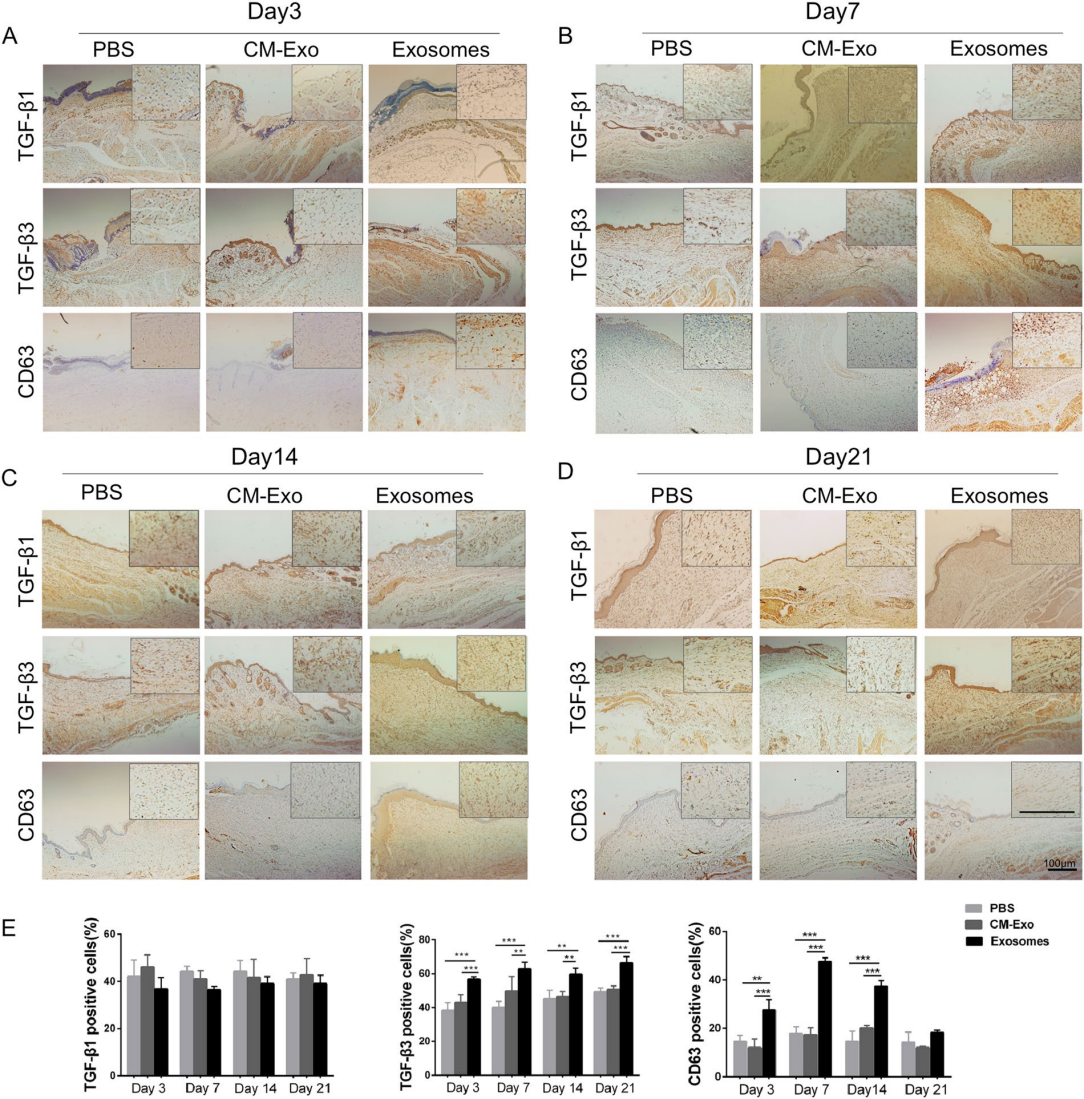
ASC-derived exosomes increased the ratio of TGF-β3 to TGF-β1 *in vivo*. (**A–D**) Representative images of immunohistochemical staining of TGF-β1, TGF-β3 and CD63 at days 3, 7, 14 and 21 following treatment with 200 µg ASC-Exos or the same volume of PBS orexosome-free conditioned medium (CM-Exo). Scale bar = 100 µm. (**E**) Analysis of the relative density of TGF-β1, TGF-β3 and CD63. Results are presented as mean ± SD; n = 5; *p < 0.05, ** p < 0.01, ***p < 0.001, compared with the control groups.

TGF-β can be produced by most cell types, including fibroblasts, keratinocytes and immune cells. TGF isoforms play key roles in all stages of wound healing. Immunohistochemical staining of wounds treated with ASC-Exos revealed that TGF-β3 positive cells were more abundant than in the PBS and CM-Exo groups at days 3, 7, 14 and 21, while the number of TGF-β1-positive cells showed little change during the process of wound healing (Fig. 3). Thus, the ASC-Exos increased the ratio of TGF-β3 to TGF-β1. To investigate the distribution of ASC-Exos in the wound area, we determined the expression of CD63, an exosomal marker, using immunohistochemical staining. We observed that the expression of CD63 was higher in the exosome-treated group, and reached a peak at day 7 (Fig. 3). The results indicate that the ASC-Exos accumulated in the wound area after they were injected into mice.

### Effects of ASC-Exos on the expression of scarless wound healing-associated genes in dermal fibroblasts

**Figure 4:**
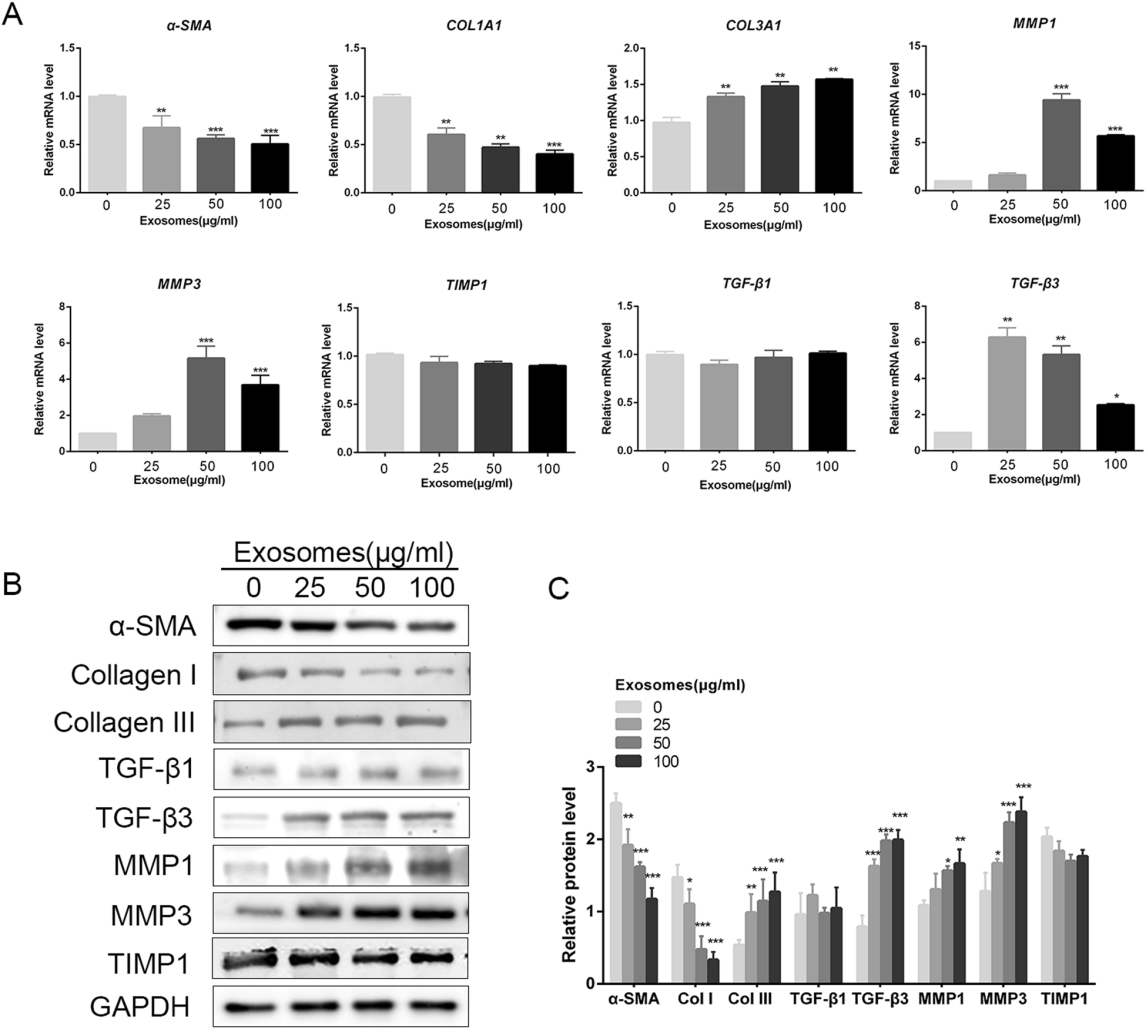
ASC-derived exosomes affected the expression of genes associated with scarless wound healing in dermal fibroblasts. (**A**) mRNA expression of *α-SMA*, *COL1A1*, *COL3A1*, *MMP1*, *MMP3*, *TIMP1*, *TGF-β1* and *TGF-β3* in human fibroblasts treated with 0, 25, 50 or 100 µg/mL ASC-Exos for 24 hours. (**B**) Western blot analysis of the expression of α-SMA, Collagen I, Collagen III, MMP1, MMP3, TIMP1, TGF-β1and TGF-β3 in fibroblasts treated with ASC-Exos (100 µg/mL) for 24 hours. (**C**) Densitometric analysis of the western blot bands. Results are presented as mean ± SD; n = 3; *p < 0.05, **p < 0.01, ***p < 0.001, compared with the control groups.

Dermal fibroblasts, the effector cells of scar formation, orchestrate both the synthesis and degradation of ECM and collagen. Our previous studies confirmed that ASC-Exos can be internalized by fibroblasts. In this study, we investigated the effects of ASC-Exos on the expression of ECM-related genes by dermal fibroblasts. As shown in Fig. 4A, after stimulation with different doses of ASC-Exos, the mRNA expression of *α-SMA* and *Col1A1* decreased, the expression of *TGF-β3*, *COL3A1*, *MMP1* and *MMP3* increased, while *TIMP1* and *TGF-β1* remained essentially unchanged (Fig. 4A). Similar to the mRNA trends, the protein levels of MMP3, COL3A1 and TGF-β3 were increased markedly in response to exosome treatment, and the levels of α-SMA and *COL1A1* were decreased, while TIMP1 and TGF-β1 showed no significant change (Fig. 4B,C). These results indicate that the ASC-Exos also increased the ratio of collagen III to collagen I *in vitro*, as well as the ratios of TGF-β3 to TGF-β1, and MMP1 and -3 to TIMP1.

### ASC-Exos activated the phosphorylation of ERK1/2 and increased the level of MMP3 via the ERK/MAPK pathway

**Figure 5:**
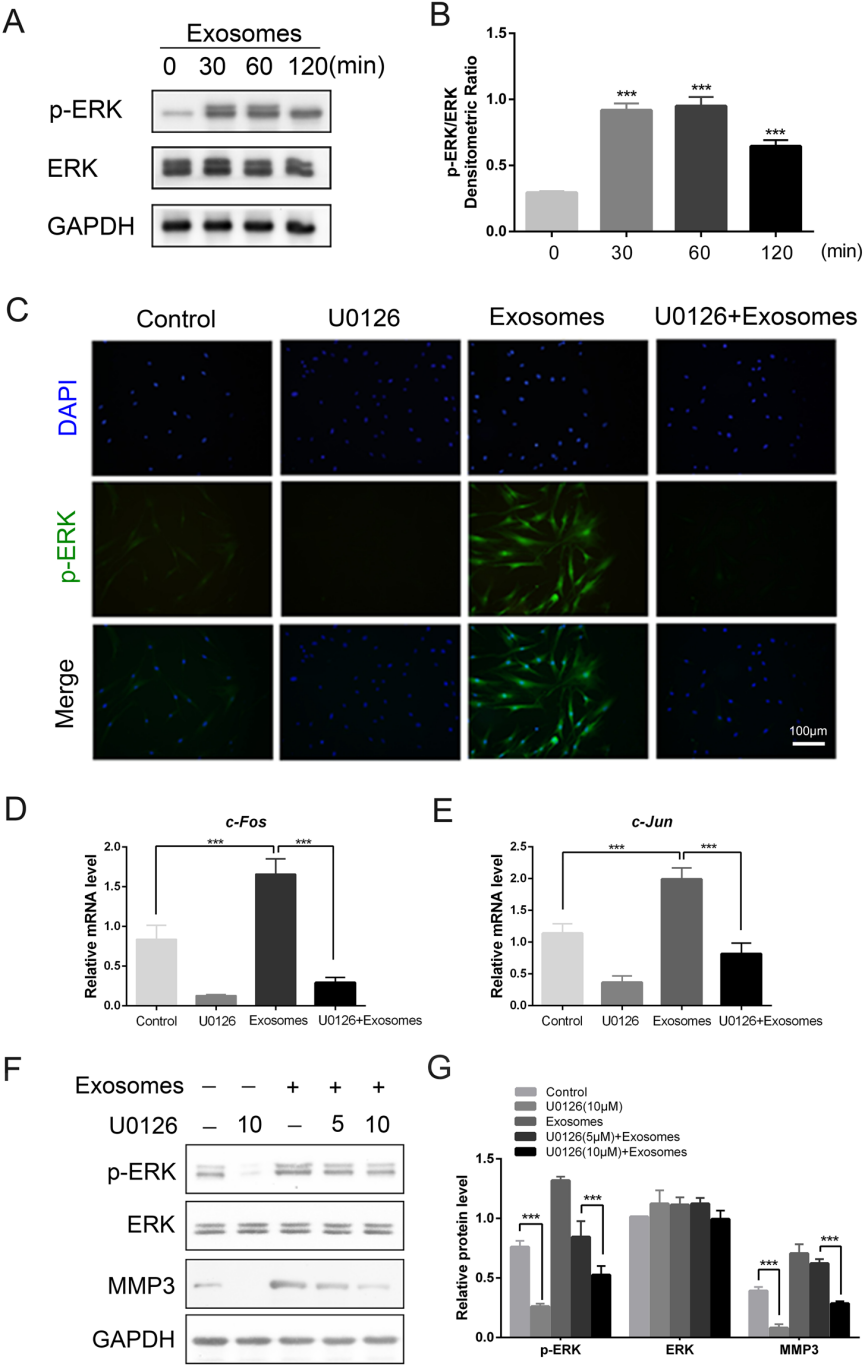
ASC-derived exosomes activated the ERK/MAPK pathway and increased MMP3 levels via this pathway. (**A**,**B**) Western blot analyses and densitometric analysis of p-ERK and ERK protein expression in fibroblasts treated with ASC-Exos (100 µg/mL) for 0, 30, 60 or 120 min (n = 3, ***p < 0.001). (**C**) Fibroblasts were treated with PBS (control), ERK inhibitor (U0126, 10 µM) or ASC-Exos for 3 hours. The nuclear translocation of p-ERK was determined by immunofluorescence staining. Scale bar = 100 µm. (**D**,**E**) mRNA expression ofdownstream genes of the ERK/MAPK pathway (*c-Jun*, *c-Fos*) in fibroblasts treated with PBS (control) or ASC-Exos in the presence or absence of U0126 (10 µM). (**F**,**G**) Western blotting and densitometric analyses of p-ERK, ERK and MMP3 protein expression in fibroblasts treated with PBS (control) or ASC-Exos in the presence or absence of U0126 (5 µM or 10 µM). Data are shown as mean ± SD; n = 3; ***p < 0.001, compared with the control groups.

To investigate the mechanisms by which ASC-Exos mediate the expression of scarless healing-related genes, we used western blot analysis to measure the protein levels of phospho-ERK1/2 (p-ERK), the activated forms of the ERK proteins, following the stimulation of fibroblasts with ASC-Exos. We found that p-ERK was rapidly activated by ASC-Exos, following 30 min of treatment (Fig. 5A,B). The analysis of immunofluorescence results suggested that ASC-Exos also induced more nuclear translocation of p-ERK in fibroblasts (Fig. 5C). Furthermore, we also observed that the expression of downstream genes of the ERK/MAPK pathway (*c-Jun*, *c-Fos*) was increased by ASC-Exos, and their increased expression was almost completely abrogated by U0126, a specific inhibitor of p-ERK (Fig. 5D,E).

In order to determine whether the ERK pathway affected the expression of scarless healing-related genes, we treated the fibroblasts with dimethyl sulfoxide or ASC-Exos in the presence or absence of U0126. The protein expression of α-SMA, MMP1, MMP3, TIMP1, TGF-β1 and TGF-β3 was then analysed by western blotting. The increases of phosho-ERK1/2 and MMP3 expression in response to treatment with ASC-exos were abrogated by U0126 in a concentration-dependent manner(Fig. 5F,G), while the other genes were not affected. Together, these data suggest that the ASC-Exos activated the ERK/MAPK signalling pathway and increased the level of MMP3 via this pathway.

## Discussion

Most previous studies have focused on exploring the wound phenotype and the mechanism of how MSC-derived exosomes promote wound healing. However, few investigations have addressed their effects on reducing scar formation and the underlying mechanisms by which they do so. In the present study, we observed that ASC-Exos reduced the extent of scarring and switched the ratio of collagen I to collagen III from a scar-promoting high ratio to an anti-scaring low ratio, similar to that seen in foetal wound healing. We also observed that exosome treatment reduced myofibroblast differentiation and decreased the ratio of TGF-β1 to TGF-β3 *in vivo*. In addition, we found that ASC-Exos activated the ERK/MAPK pathway in skin dermal fibroblasts *in vitro*, and increased the level of MMP3 via activation of the ERK pathway, thereby increasing the ratio of MMP3 to TIMP1 and regulating the remodelling of ECM.

A significant proportion of scars are classified as pathological, and these cause considerable problems. As yet, no permanent and consistently effective treatment for scars with few side effects is available. Cutaneous scar formation is a consequence of the wound-healing process. It involves the orchestration of a complex sequence of interactions between cells, ECM components and signalling molecules. The proliferative and remodelling phases of wound healing are closely connected with the production and reorganization of ECM, which is crucial in determining the extent of scarring. Scar tissue is characterized by the exaggerated deposition of ECM components and the lack of skin appendages such as hair follicles and sweat glands. The ECM consists of collagens, fibronectins, proteoglycans, laminins, elastins, hyaluronan and glycoproteins. Among these, collagens are the most abundant components of the developing ECM. ECM remodelling, especially collagen synthesis and degradation, is the key cellular and molecular event contributing to scarring. Interestingly, scarless healing occurs in the early or midgestation stages of embryonic development, and the differences between foetal wound healing and adult wound healing have important implications for reducing scar formation. In foetal wound tissue, the ratio of collagen type III to type I is higher and the arrangement of collagen is finely reticular with less cross-linking. There is also a higher ratio of TGF-β3 to TGF-β1 and of MMPs to TIMPs.

Recent studies have focused on the beneficial effects of mesenchymal stem cells on tissue engineering, including their recruitment of host cells and release of secretory factors and matrix proteins. Much evidence indicates that adipose stem cells (ASCs) are a potential stem cell source for regenerative medicine^32^ because of their advantages of convenient acquisition and regenerative potential. They can be cultured in large quantities from adipose tissues, which are easily harvested with little discomfort for the donors. However, most MSCs would not be viable within a short time after transplantation into a patient. Immunological rejection and chromosomal variation always restrict the clinical application of stem cells^33^. Hence, many researchers have turned their attention to exosomes, which are a secretory product of MSCs. Owing to their lack of immune rejection, high stability, and easily controlled dosage and concentration, exosomes have attracted increasing attention in the wound healing field^34–36^. Our histological analysis of full wound sections further demonstrated that exosome treatment can reduce scar width and depth. Some studies have suggested that exosomes promote collagen synthesis^37^, while others have indicated that exosomes suppress collagen deposition. These controversial results may be attributed to the double role of collagen and the complex function of exosomes during different stages of wound healing. Promoting collagen synthesis in the early stage of wound healing is necessary. On the other hand, overaggressive collagen deposition results in scarring. Our previous study found that exosomes promote collagen synthesis, while in the late stage of wound healing, they might inhibit collagen synthesis^21^. However, no previous studies have reported the effects of exosomes on the ratio of collagen III to collagen I and the arrangement of collagen fibres. Our study demonstrated that, after exosome treatment, the amount of collagen III in the wound bed increased, and it was arranged in a fine reticular pattern. These results indicate that ASC-Exos regulate the deposition and arrangement of collagen to promote a scarless pattern.

While the mechanisms of scarless wound healing are not completely understood, it has become clear that TGF-β3/TGF-β1, myofibroblasts and MMPs/TIMPs play a critical role in the process of wound healing. Our data demonstrated that the expression of MMP1 and MMP3 was elevated in response to exosome treatment, but the level of TIMP1 remained unchanged. Many studies have reported that TGF-β isoforms are important factors involved in ECM deposition and scar formation. TGF-β3 has been used as a scar prevention and reduction therapeutic^38^ and acts as a negative regulator of the myofibroblastic phenotype, while TGF-β1 promotes myofibroblast differentiation and granulation tissue formation^39,﻿40^. Ourimmunohistochemical examination and western blot analysis showed an increased ratio of TGF-β3 to TGF-β1. It has been reported that myofibroblasts are embedded in the ECM that they secrete, and that they also secrete MMPs and TIMPs to remodel this ECM. Consistent with our *in vivo* experiments, we found that ASC-Exos substantially increased the expression of TGF-β3 and collagen III, but decreased the expression of α-SMA and collagen I in dermal fibroblasts (Fig. 4). Exosome treatment also increased the ratio of MMPs to TIMP1 (Fig. 4). In addition, ASC-Exos activated the ERK signalling pathway in fibroblasts and elevated the expression of MMP3 through this pathway.

**Figure 6:**
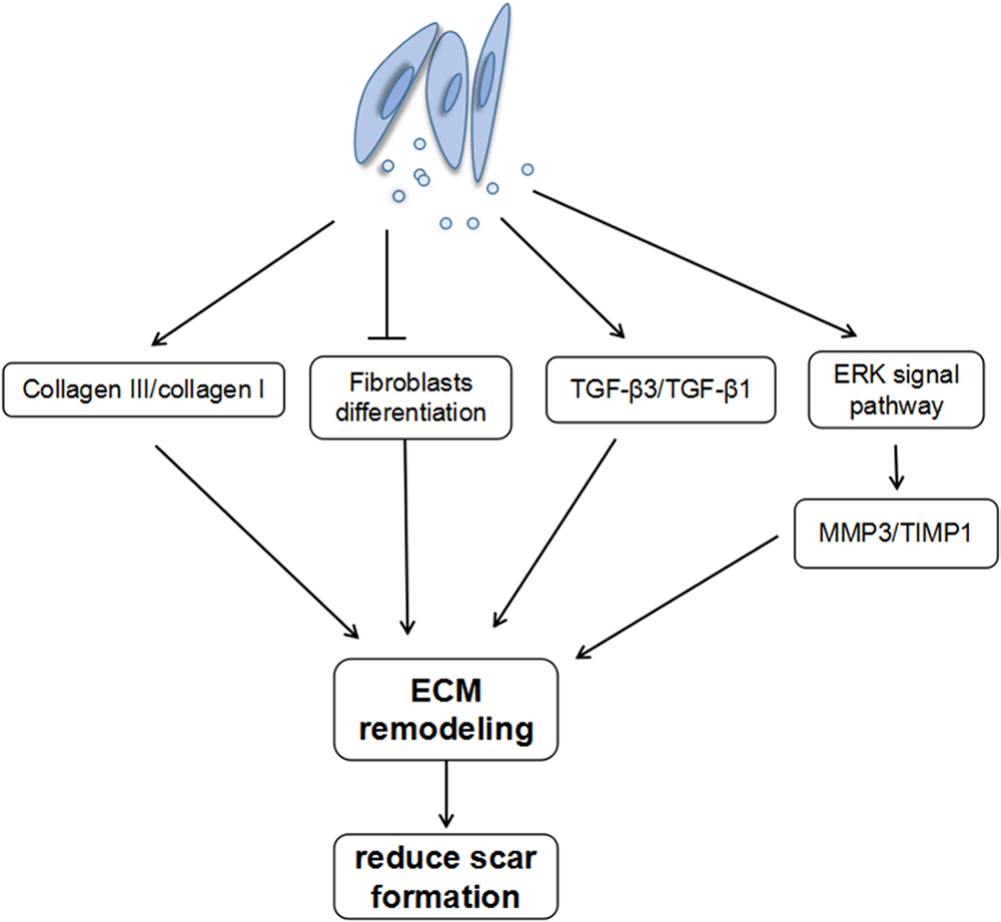
ASC-derived exosomes contributed to regulating ECM reconstruction and reducing scar formation. Exosomes increased the ratio of collagen III to collagen I, reduced myofibroblast differentiation and increased the ratio of TGF-β3 to TGF-β1 *in vivo*. In addition, exosomes activated the ERK/MAPK pathway in skin dermal fibroblasts and increased the level of MMP3 via activation of the ERK pathway, thereby increasing the ratio of MMP3 to TIMP1 and regulating ECM remodelling.

In conclusion, we provide evidence that ASC-Exos can promote ECM remodelling and scarless repair in a mouse skin incision model. The underlying mechanism may involve the regulation of ECM molecules and the prevention of myofibroblast differentiation. Furthermore, the ERK/MAPK pathway is likely to play a significant role in the positive effects of ASC-Exos on scar reduction (Fig. 6). Together with our previously published study^21^, these results indicate that ASC-Exos are a promising therapeutic approach for enhancing wound healing and preventing scars.

## Materials and Methods

This study was approved by the Committee of Ethics of Wuhan Union Hospital and the Institutional Research Ethics Committee of Tongji Medical College, Huazhong University of Science and Technology (Wuhan, China). The protocols, including all relevant details, were performed in accordance with the Ethics Committee of Tongji Medical College requirements (IORG No: IORG0003571; the statement of ethics is in the Supplementary Information). We can confirm that all methods were performed in accordance with the relevant guidelines and regulations.

### Cell culture

Human ASCs were obtained using a mechanical and enzymatic method as previously described^41^. Human subcutaneous adipose tissue samples were acquired from healthy mothers (18–30 years old) undergoing caesarean section, with informed consent. The adipose tissue was washed several times in sterile phosphatebuffered saline (PBS). Next, the samples were cut into small pieces and digested with collagenase type I (0.1%, Gibco) at 37 °C for 100 min. After filtering through a 100 µm mesh filter, a cell suspension was obtained and centrifuged at 800 RCF for 5 min. The cells were cultivated in specific MSC culture medium (Cyagen) at 37 °C in 5% CO_2_. ASCs frompassages 1–6 were used in all experiments.

Primary cultures of dermal fibroblasts were established using procedures described previously^42^. Human foreskins were washed several times with PBS. Theunderlying fat was removed and the tissue was treated with dispase II (4 mg/mL; Gibco) overnight at 4 °C to separate epidermis and dermis. The dermis was then cut into smallpieces and digested with collagenase type I (0.1%; Gibco) for 3 hours at 37 °C to isolate fibroblasts. Fibroblasts were cultured in high-glucose Dulbecco’s modified Eagle’s medium (DMEM; Thermo Fisher) containing 10% foetal bovine serum (FBS; Thermo Fisher) at 37 °C with 5% CO_2_. When cells reached 70% confluence, they were trypsinized and subcultured to culture flasks.

### Exosome isolation

ASCs at passage 3 were cultured until cells reached 70–80% confluency, and then the medium were replaced with serum-free DMEM/F12 for 24 hours. The conditioned medium (CM) was collected and centrifuged at 3000 RCF for 15 min to remove cells and cell debris. Supernatants were passed through a 100 kDa molecular weight Amicon® Ultra-15 Centrifugal Filter Device (Millipore) and were concentrated to 1–2 mL. The filtrate was also collected, passed through a 0.22 µm filter, and stored at −70 °C (exosome-free CM, CM-Exo). The appropriate volume of ExoQuick-TC (System Biosciences) was added to the concentrated CM, it was mixed well by inverting the tube, and then refrigerated overnight (for at least 12 hours) at 4 °C. The ExoQuick-TC/CM mixture was then centrifuged at 1500 RCF for 30 min. The exosome-enriched pellets were resuspended in PBS (approximately 100–200 µL) and then passed through a 0.22 µm filter. The exosome concentrations were measured using a BCA protein assay kit (Beyotime Institute of Biotechnology).

The presence of the ASC-Exos was subsequently confirmed by using a NanoSight LM10 instrument (Nanosight) and Nanoparticle Tracking Analysis software version 2.2 (NanoSight), and by detection of the exosomal surface markers, CD9 (Abcam) and CD63 (Abcam) using western blot analysis.

### Animal model

Balb/c mice (6–8 weeks old) were purchased from the Animal Centre of Disease Control and Prevention. The skin wound model was established as previously described^21^. Animals were anesthetized with sodium pentobarbital (30 mg/kg), and then their backs were shaved. Full-thickness dorsal wounds of uniform size (approximately 2.0 × 1.5 cm^2^) were made on the shaved skin. On the second day after surgery, the animals were randomly divided into three groups. The groups received intravenous injections of PBS (200 µL), CM-Exo (200 µL), or ASC-Exos (200 µg suspended in 200 µL PBS). Mice were sacrificed for further analysis at the indicated times.

### Histological analysis and immunohistochemistry

Wound samples were harvested individually at day 3, 7, 14 and 21 after wounding, fixed in paraformaldehyde (4%, pH 7.4), and then progressively dehydrated and embedded in paraffin. H&E staining and picrosirius red staining were used for histological observations^43^. Following picrosirius red staining, collagen I displays astrong orange-red birefringence under polarized light, whereas collagen III has a weak blue-green birefringence^31,44,45^. For immunohistochemistry, paraffin sections were dewaxed and nonspecific binding was blocked with bovine serum albumin (1%) for 30 min. Samples were incubated with primary antibodies against CollagenI(1:100, Abcam), Collagen III(1:100, Abcam), TGF-β1 (1:200, Proteintech), TGF-β3 (1:200, Proteintech) and CD63 (1:200, Proteintech) overnight at 4 °C. After the wash steps and incubation with secondary antibodies, the sections were incubated with diaminobenzidine substrate and counterstained with haematoxylin. Immunopositive cells were counted using Image-Pro Plus software.

### qRT-PCR

Skin wound tissues or fibroblasts were harvested after treatment to analyse the mRNA expression levels of genes associated with wound healing by qRT-PCR. Total RNA was extracted using an RNeasy Mini Kit (Qiagen) according to the manufacturer’s instructions, and then reversed-transcribed into complementary DNA.qRT-PCR was performed with SYBR Green PCR reagent on an ABI Prism 7300 system (Applied Biosystems). The primer sequences are listed in Table 1. The reaction conditions were 30 s at 95 °C, followed by 40 cycles of 95 °C for 5 s and 60 °C for 30 s. The relative expression levels were calculated using the comparative CT method (ΔΔCT). *GAPDH* was used as an internal control.

**Table 1:** Primers of quantitative reverse transcription–polymerase chain reaction (qRT-PCR).

Gene	Forward primer (5′ to 3′)	Reverse primer (5′ to 3′)
*α-SMA*	GCTACTCCTTCGTGACCACAG	GCCGTCGCCATCTCGTTCT
*MMP1*	GGCTGAAAGTGACTGGGAAACC	TGCTCTTGGCAAATCTGGCGTG
*MMP3*	CTGGACTCCGACACTCTGGA	CAGGAAAGGTTCTGAAGTGACC
*TIMP1*	TTCTGCAATTCCGACCTCGTCATC	ATCCCCTAAGGCTTGGAACCCTTT
*TGF-β1*	AGTTGTGCGGCAGTGGTTGA	GCCATGAATGGTGGCCAGGT
*TGF-β3*	ACTTGCACCACCTTGGACTTC	GGTCATCACCGTTGGCTCA
*GAPDH*	GGCACAGTCAAGGCTGAGAATG	ATGGTGGTGAAGACGCCAGTA
*COL1A1*	ATCAACCGGAGGAATTTCCGT	CACCAGGACGACCAGGTTTTC
*COL3A1*	GCCAAATATGTGTCTGTGACTCA	GGGCGAGTAGGAGCAGTTG
*Gapdh*	AGGAGCGAGACCCCACTAACA	AGGGGGGCTAAGCAGTTGGT
*Col1a1*	CTGGCGGTTCAGGTCCAAT	TTCCAGGCAATCCACGAGC
*Col3a1*	CAAGGGTGATCGTGGTGAAAA	CCAGGGAATCCTCGATGTCCT

### Western Blotting

After various treatments, the whole wound tissue or cells were washed with ice-cold PBS and extracted in lysis buffer containing a protease inhibitor cocktail (Thermo Scientific). The protein concentration of each sample was determined using a BCA protein assay kit. Total protein extract (30 µg) was resolved by 10% sodium dodecylsulfate polyacrylamidegel electrophoresis and transferred to polyvinylidenedifluoride membranes. The membranes were blocked with nonfat milk (5%) in tris-buffered saline–Tween-20(TBS-T;10 mM Tris, 150 mM NaCl, 0.1% Tween-20) for 1 hour at room temperature; they were then washed several times with TBS-T and subsequently incubated with primary antibodies at 4 °C overnight. The primary antibodies used included the following: anti-p-ERK1/2 (1:2000, Cell Signaling Technology), anti-ERK1/2 (1:2000, Cell Signaling technology), anti-MMP1 (1:1000, Abclonal), anti-MMP3 (1:1000, Proteintech), anti-TGF-beta1 (1:1000, Abclonal), anti-α-SMA (1:500, Proteintech), anti-TGF-beta3 (1:500, Proteintech), anti-TIMP1 (1:1000, Santa cruz), anti-collagen I (1:1000, Abcam), and anti-collagen III (1:1000, Abcam). An anti-GAPDH antibody (1: 8000, Proteintch) was used as an internal control. After washing three times with TBS-T, membranes were incubated with the appropriate secondary antibodies for 1 h at room temperature. The membranes were developed with an enhanced chemiluminescence detection system and exposed to X-ray film. Immunoreactive bands were quantified using Image Analyzer software.

### Immunofluorescence

Excised tissues or cells were fixed in paraformaldehyde (4%). Immunofluorescence analysis was conducted using the following antibodies: rabbit anti-α-SMA (1:200, Proteintech), or rabbit anti-p-ERK1/2 (1:2000, Cell Signaling Technology). After washing with PBS, labelling was visualized using an Alexa Fluor 594-conjugated goat anti-rabbit antibody (1:200) or an Alexa Fluor 488-conjugated goat anti-rabbit antibody (1:200), and the nuclei were counterstained with 4,6-diamidino-2-phenylindole (DAPI). Images were acquired using a fluorescence microscope with the appropriate excitation illumination.

### Statistical analyses

All data are shown as means ± standard deviations (SD). Data were analysed using Student’s one-way or two-way analysis of varianceto compare the differences between groups. Statistical analysis was performed using GraphPad Prism 6 software. P values < 0.05 were considered statistically significant.

## Supplementary information

Supplementary information.
