# Author Correction: Exosomes secreted by human adipose mesenchymal stem cells promote scarless cutaneous repair by regulating extracellular matrix remodelling

**DOI:** 10.1038/s41598-018-24991-y

**Published:** 2018-05-01

**Authors:** Lu Wang, Li Hu, Xin Zhou, Zehuan Xiong, Chenguang Zhang, Hassan M. A. Shehada, Bo Hu, Jinlin Song, Lili Chen

**Affiliations:** 1 Department of Stomatology, Union Hospital, Tongji Medical College, Huazhong University of Science and Technology, Wuhan, 430022 China; 2 College of Stomatology, Chongqing Medical University, Chongqing, 401147 P.R. China

Correction to: *Scientific Reports* 10.1038/s41598-017-12919-x, published online 17 October 2017

In the original version of this Article, there were errors in Affiliation 1 which was incorrectly listed as ‘Department of Stomatology, Union Hospital, Tongji Medical College, Huazhong University of Science and Technology, Wuhan, Hubei, 430022, China.’ The correct affiliation is listed below:

Department of Stomatology, Union Hospital, Tongji Medical College, Huazhong University of Science and Technology, Wuhan 430022, China.

These errors have now been corrected in the PDF and HTML versions of the Article.

