# Author Correction: Exosomes secreted by human adipose mesenchymal stem cells promote scarless cutaneous repair by regulating extracellular matrix remodelling

**DOI:** 10.1038/s41598-021-82225-0

**Published:** 2021-02-01

**Authors:** Lu Wang, Li Hu, Xin Zhou, Zehuan Xiong, Chenguang Zhang, Hassan M. A. Shehada, Bo Hu, Jinlin Song, Lili Chen

**Affiliations:** 1 Department of Stomatology, Union Hospital, Tongji Medical College, Huazhong University of Science and Technology, Wuhan, 430022 China; 2 College of Stomatology, Chongqing Medical University, Chongqing, 401147 P.R. China

Correction to: *Scientific Reports* 10.1038/s41598-017-12919-x, published online 17 October 2017

The original version of this Article contained errors in Figure 3B, where the incorrect image was provided for the TGF-β1/CM-Exo panel. In addition, in Figure 3D the images for the TGF-β1/PBS and TGF-β1/Exosomes panels were incorrect. The original version of Figure 3 appears below as Figure 1.

**Figure 1:**
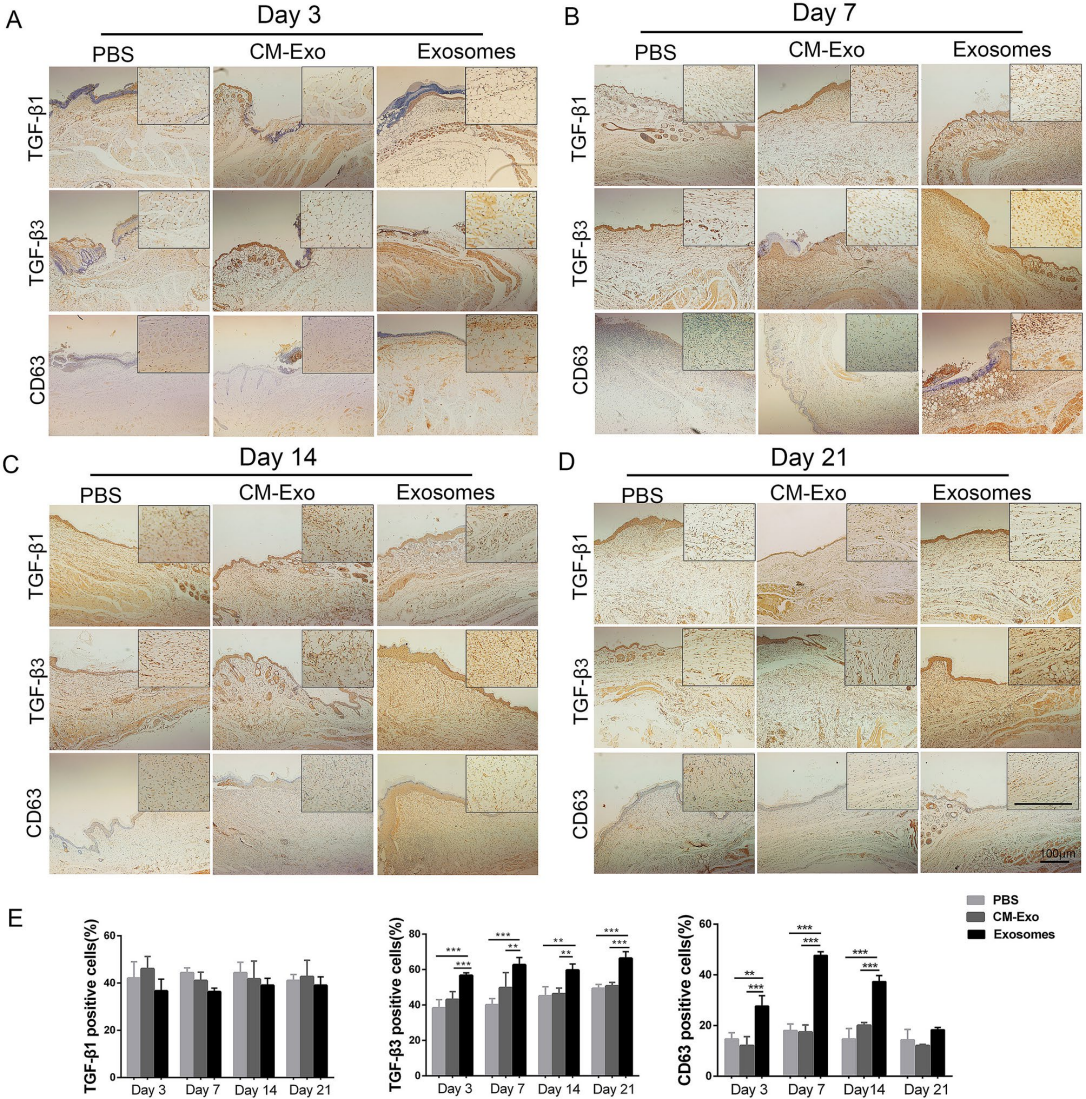
The original incorrect version of Figure 3.

Furthermore, Figure 4B contained an error, where the original representative TIMP-1 blot was not the best match for the quantitative analysis. The original version of Figure 4 appears below as Figure 2.

**Figure 2:**
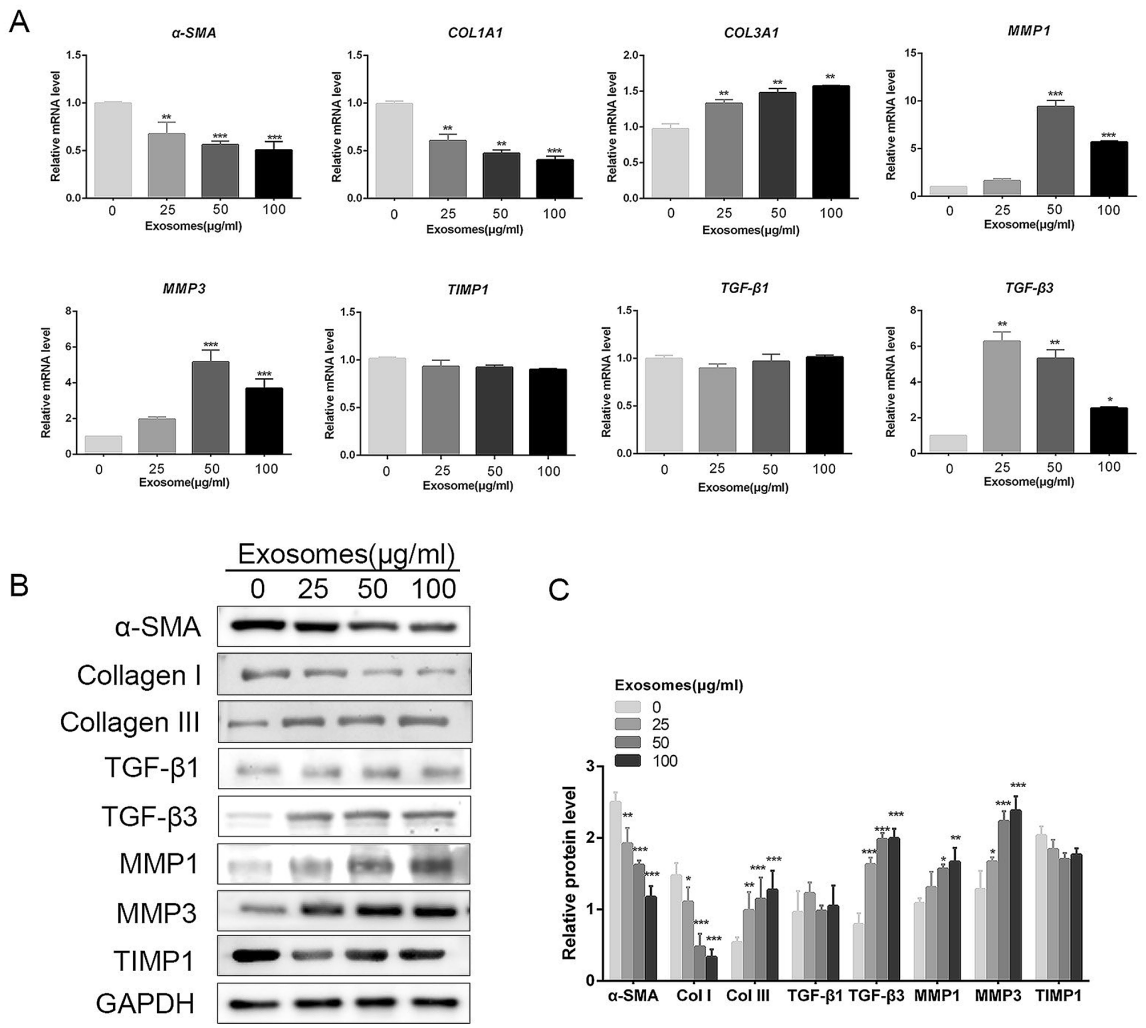
The original incorrect version of Figure 4.

Finally, the full blots were not provided in the original Article, these have now been provided as a Supplementary Information file that is linked to this correction notice.

The changes do not affect the overall conclusions of the Article. These errors have now been corrected in the PDF and HTML versions of the Article.

## Supplementary Information

Supplementary information.

