# Supplementary material for: Exosomes secreted by human adipose mesenchymal stem cells promote scarless cutaneous repair by regulating extracellular matrix remodelling
Source: Sci Rep. 2017 Oct 17;7:13321. doi: 10.1038/s41598-017-12919-x (PMC5645460; doi:10.1038/s41598-017-12919-x)
Supplement: Supplementary file 1 — Supplementary information. [file 41598_2017_12919_MOESM1_ESM.pdf]

Exosomes secreted by human adipose mesenchymal stem cells promote scarless cutaneous repair by regulating extracellular matrix remodeling

LuWang[1], Li Hu[1], Xin Zhou[1], Zehuan Xiong[1], Chenguang Zhang[1], Hassan M. A. Shehada[1], Bo Hu[2], Jinlin Song[2] & Lili Chen[1]

[1] Department of Stomatology, Union Hospital, Tongji Medical College, Huazhong University of Science and Technology, Wuhan, Hubei, 430022, China.
[2] College of Stomatology, Chongqing Medical University, Chongqing, 401147, P.R. China.

Lu Wang and Li Hu contributed equally to this work. Correspondence and requests for materials should be addressed to J.S. (email: soongjl@163.com) or L.C. (email: lily-c1030@163.com)

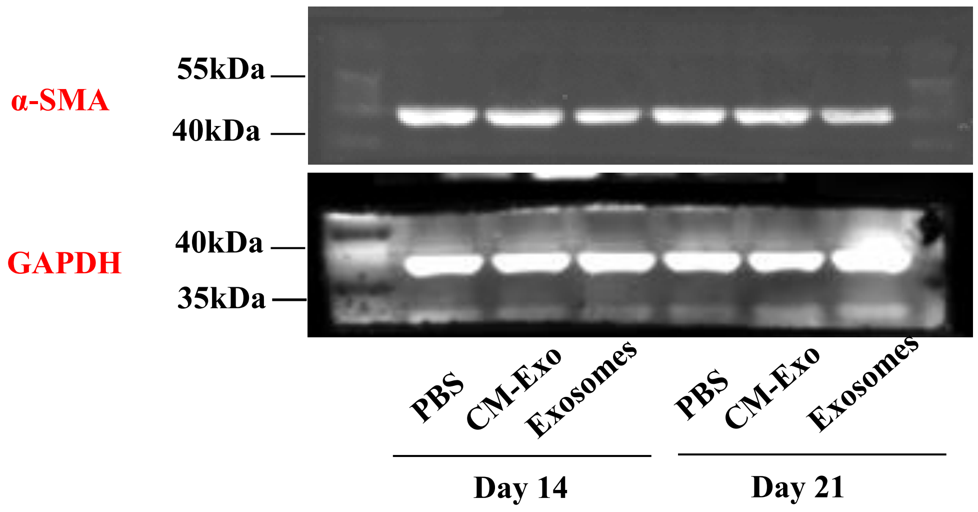

Supplementary Figure 1. Full blots for the expression of α-SMA in Figure 2E

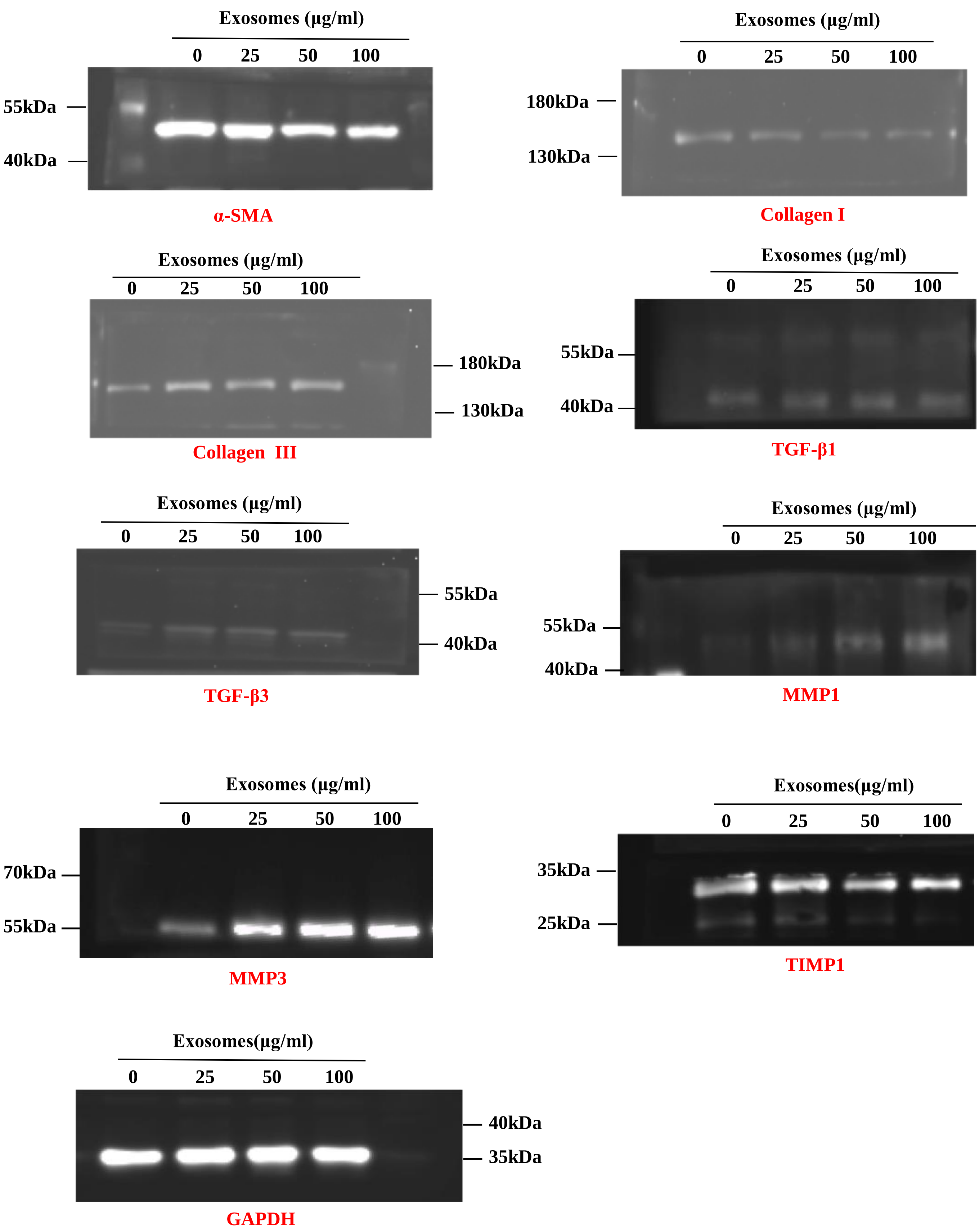

Supplementary Figure 2. Full blots for the expression of several proteins in fibroblasts treated with ASC-Exos for 24 hours in Figure 4B

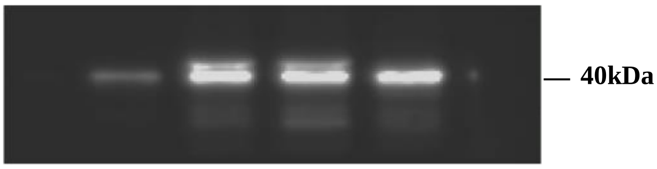

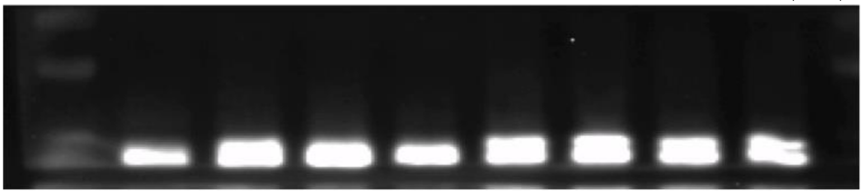

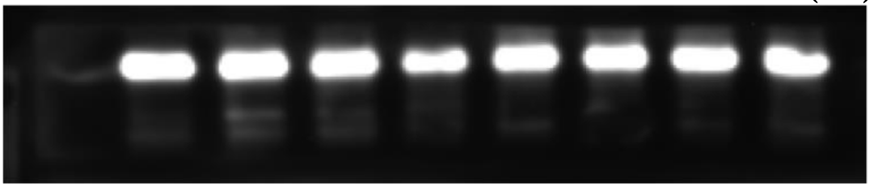

Supplementary Figure 3. Full blots for the expression of p-ERK and ERK in Figure 5A

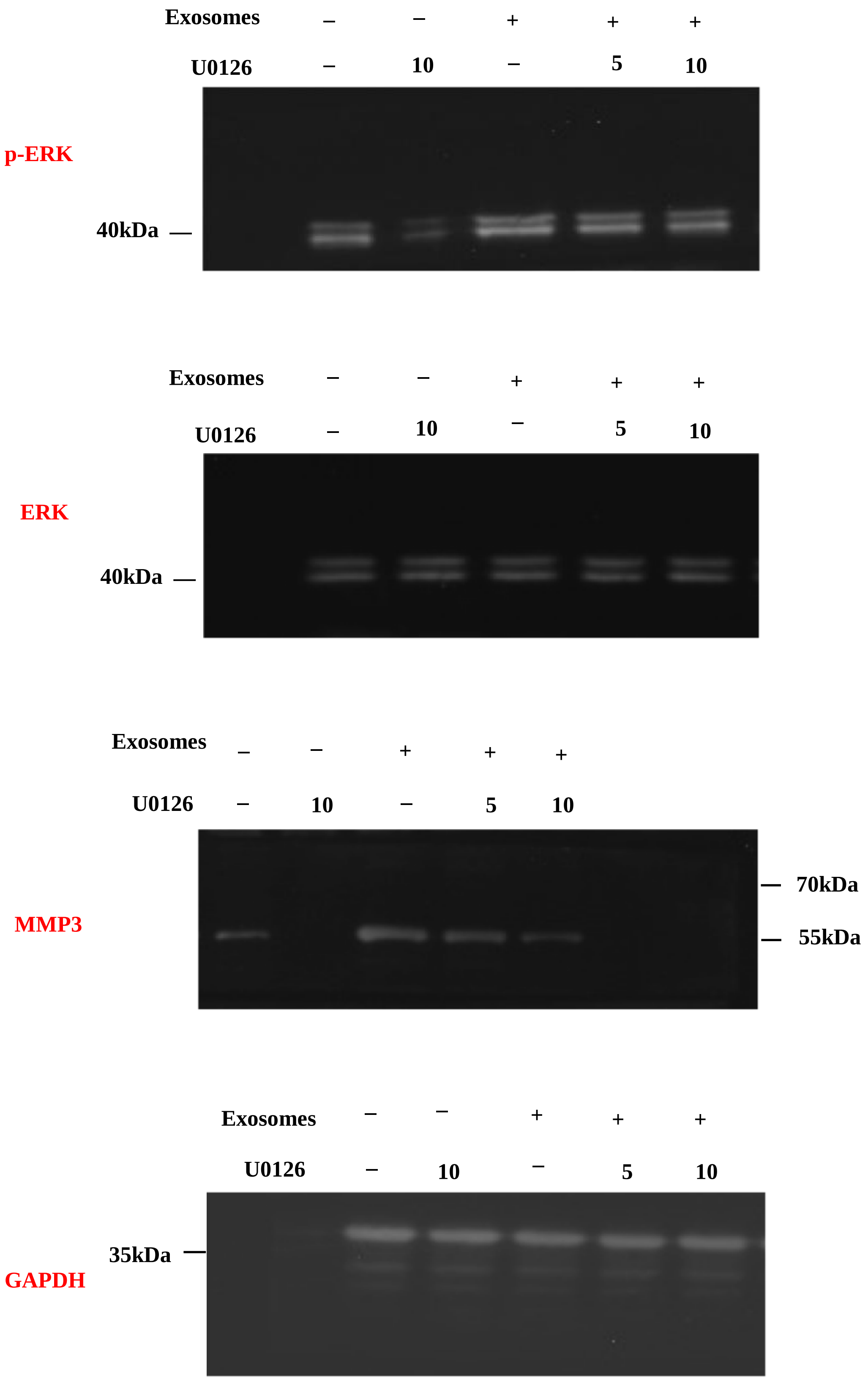

Supplementary Figure 4. Full blots for the expression of p-ERK, ERK and MMP3 in Figure 5F
